# Repair of Abdominal Wall Defects with Biodegradable Laminar Prostheses: Polymeric or Biological?

**DOI:** 10.1371/journal.pone.0052628

**Published:** 2012-12-21

**Authors:** Gemma Pascual, Sandra Sotomayor, Marta Rodríguez, Bárbara Pérez-Köhler, Juan M. Bellón

**Affiliations:** 1 Department of Surgery, Networking Research Center on Bioengineering, Biomaterials and Nanomedicine (CIBER-BBN), Faculty of Medicine, Alcalá University, Alcalá de Henares, Madrid, Spain; 2 Department of Medical Specialties, Networking Research Center on Bioengineering, Biomaterials and Nanomedicine (CIBER-BBN), Faculty of Medicine, Alcalá University, Alcalá de Henares, Madrid, Spain; Harvard Medical School, United States of America

## Abstract

**Introduction:**

Biological and synthetic laminar absorbable prostheses are available for the repair of hernia defects in the abdominal wall. They share the important feature of being gradually degraded in the host, resulting in place the formation of a neotissue. This study was designed to assess the host tissue’s incorporation of collagen bioprostheses and a synthetic absorbable prosthesis.

**Methods:**

Partial defects were created in the abdominal walls of 72 New Zealand rabbits and repaired using collagen bioprostheses Tutomesh® and Strattice® or a synthetic prosthesis Bio-A®. Specimens were collected for light microscopy, collagens gene and protein expression, macrophage response and biomechanical resistance at 14, 30, 90 and 180 days post-implantation.

**Results:**

Tutomesh® and Bio-A® were gradually infiltrated by the host tissue and almost completely degraded by 180 days post-implantation. In contrast, Strattice® exhibited material encapsulation, no prosthetic degradation and low cell infiltration at earlier timepoints, whereas at later study time, collagen deposition could be observed within the mesh. In the short term, Bio-A® exhibited higher level of collagen 1 and 3 mRNA expression compared with the two other biological prostheses, which exhibited two peaks of higher expression at 14 and 90 days. The expression of collagen III was homogeneous throughout the study and collagen I deposition was more evident in Strattice®. Macrophage response decreased over time in biomeshes. However, in the synthetic mesh remained high and homogeneous until 90 days. The biomechanical analysis demonstrated the progressively increasing tensile strength of all biomaterials.

**Conclusions:**

The tissue infiltration of laminar absorbable prostheses is affected by the structure and composition of the mesh. The synthetic prosthesis exhibited a distinct pattern of tissue incorporation and a greater macrophage response than did the biological prostheses. Of all of the laminar, absorbable biomaterials that were tested in this study, Strattice® demonstrated the optimal levels of integration and degradation.

## Introduction

The study and development of prosthetic materials for the repair of abdominal wall defects has evolved and progressed during the past several years with the ultimate goal of discovering the “ideal prosthesis.”

The classic polymeric materials (such as polyester, polypropylene and expanded polytetrafluoroethylene), despite providing satisfactory results, have been replaced by materials of natural origin, the latter mainly from animal sources. These implants, called “biomeshes,” which are primarily composed of collagen, can not only repair but can also regenerate new tissue that is similar to that of the human recipient [1]. During this process, the biomeshes undergo a progressive degradation in the host. Two types of materials exist: those with cross-links that stabilize the collagen molecule, thus preventing its rapid degradation, and those noncrosslinked, which undergo a progressive and variable degradation over time [2]. Clearly, the process for which these prostheses are designed is not feasible for the majority of the synthetic polymeric prostheses, which remain for life in the recipient organism; in certain instances, these synthetic prostheses elicit inflammatory and foreign body reactions with the potential for more diverse post-implant complications [3].

The advantage of using biomeshes is that the repair mechanisms approach optimal conditions. However, there may also be inconveniences, including adverse effects that have been described after implantation [4], [5]. One of the areas for improvement and research is the control of the prosthetic degradation times, particularly of noncross-linked prostheses.

Alternatively, polymeric, midterm biodegradable materials have emerged, which are indicated more for reinforcement than for tissue replacement. These materials constitute a commitment to the future within the scope of new prosthetic developments. *Bio-A*® is composed of polyglycolic acid and trimethylene carbonate. These polymers are widely known for their biocompatibility and have been predominantly used in sutures. Prior experience with this prosthesis is very limited; one *in vitro* study compares its elicited immune reaction in humans with those of biological prostheses [6], and another study relates to hernia repair [7].

Considering the hypothesis that biodegradable synthetic laminar prostheses provide advantages over certain collagen biomeshes, our objective was to study the behavior of this new prosthetic material. Noting its biodegradation characteristics (3–6 months), we compared the synthetic laminar prosthesis with collagen noncross-linked bioprostheses; our goal was to evaluate the repair and/or regenerative capacity at the receptor-tissue level in a partial abdominal wall defect model.

## Materials and Methods

### Experimental Animals

The experimental animals included 72 male New Zealand White rabbits (weighing approximately 2500 g). This study was carried out in strict accordance with the recommendations in the Guide for the Care and Use of Laboratory Animals of the National and European Institutes of Health (Spanish law 32/2007, Spanish Royal Decree 1201/2005, European Directive 2010/63/UE and European Convention of the Council of Europe ETS123). All the procedures were performed at the Animal Research Center of Alcalá University. The protocol was approved by the Committee on the Ethics of Animal Experiments of the University of Alcalá (registered code: ES280050001165).

### Prosthetic Materials

**Figure 1:**
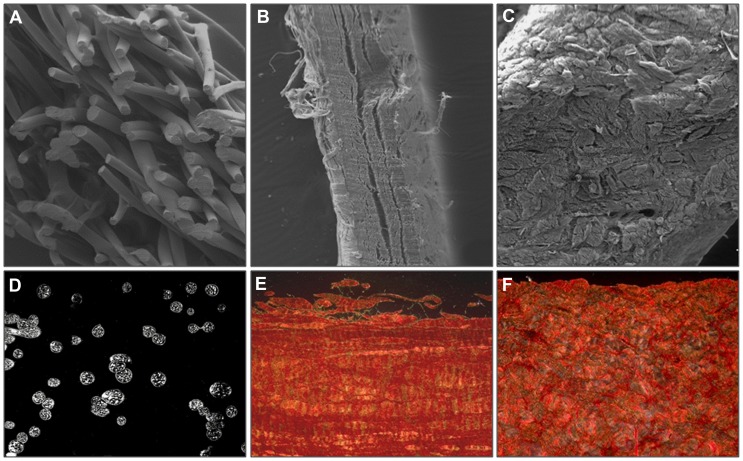
Used biomaterials. Scanning electron microscopy images (100×) showing the aspect and the thickness of *Bio-A* (A), *Tuto* (B) and *St* (C). Polarized light images, with collagen fibers displayed in red after Sirius Red staining (200×). *Bio-A* (D), *Tuto* (E) and *St* (F).

The following biomaterials were used. Characterization, before the implant was performed by scanning electron microscopy and Sirius red staining (Fig. 1):

*Bio-A® (Bio-A)* (W.L. Gore, Flagstaff, AZ, USA): a synthetic laminar bioabsorbable material (1.3 mm thick), composed of polyglycolic acid:trimethylene carbonate (PGA:TMC) fibers.
*Tutomesh® (Tuto)* (Tutogen Medical GmbH, Nümberg, Germany): a xenogenic collagen I membrane (0.5 mm) from bovine pericardium (noncross-links).
*Strattice® (St)* (LifeCell Corporation, Branchburg, NJ, USA): a porcine dermal, biological noncross-linked tissue matrix (1.2 mm thick).

### Surgical Technique

To minimize pain, all of the animals were administered 0.05 mg/kg buprenorphine (Buprecare®, Divasa Farmavic, Barcelona, Spain) 1 hour before and 3 days after the surgical procedure. Anesthesia was induced with a mixture of ketamine hydrochloride (Ketolar, Parke-Davis, Spain) (70 mg/kg), diazepam (Valium, Roche, Spain) (1.5 mg/kg) and chlorpromazine (Largactil, Rhone-Poulenc, Spain) (1.5 mg/kg) administered intramuscularly.

Using a sterile surgical technique and after making an approximately 6-cm-long incision in the skin, 3×3-cm defects were created in the lateral wall (right side) of the abdomen, transecting the planes of the external and internal oblique muscles and sparing the transversalis muscle and parietal peritoneum. The defects were then repaired by fixing a mesh of the same size to the edges of the defect, using a 4/0 polypropylene running suture interrupted at the four corners. The skin was closed using a 3/0 polypropylene running suture.

During all the study, the animals were visually inspected for signs of dehiscence of the skin wound, seroma formation, wound infection and/or mesh incompatibility.

### Experimental Design

A total of 72 implants, divided into three study groups, were made:

*Bio-A*® (n = 24): synthetic laminar bioabsorbable material, composed of PGA:TMC fibers.
*Tutomesh*® (n = 24): xenogenic collagen I membrane from bovine pericardium.
*Strattice*® (n = 24): porcine dermal, biological tissue matrix.

The animals were sacrificed following the protocols for experimental animal euthanasia, in a CO_2_ chamber at 14, 30, 90 and 180 days after implant. Study times were established with the objective of carrying out a proper follow-up of the evolution of the prosthesis, once implanted in the animal, from short to long term. Specimens of each prosthesis with some surrounding host tissue, were obtained for the different analysis. From each sample, one 1.5 cm wide and 5 cm long strip (taking the 3 cm of the sample and an additional 1 cm on each side of receptor tissue), were used for biomechanical studies. The rest of the sample was used for histology and molecular biology.

### Morphological Analyses

For light microscopy, the specimens were stained with Massońs trichrome (Goldner-Gabe) staining and examined under a Zeiss light microscope (Carl Zeiss, Oberkochen, Germany).

### RNA Isolation and Real-time PCR (RT-PCR)

Tissue fragments that were 1 cm^2^ in size were obtained from the implant area and stored at −80°C until use. The RNA was extracted by guanidine-phenol-chloroform isothiocyanate procedures using TRIzol (Invitrogen, Carlsbad, CA, USA). The RNA was recovered from the aqueous phase by precipitation. The amount and purity were measured using the optical density at 260/280 nm and 260/230 nm in a NanoDrop ND-1000 spectrophotometer (Thermo Fisher Scientific Inc., DE, USA).

Complementary DNA was synthesized from 200 ng of the total RNA by reverse transcription (RT) with oligo dT primers (Amersham, Fairfield, USA) and the M-MLV reverse transcriptase enzyme (Invitrogen). The cDNAs were amplified using the following primers: collagen 1 (col 1) (sense 5′-GAT GCG TTC CAG TTC GAG TA-3′ and antisense 5′-GGT CTT CCG GTG GTC TTG TA-3′), collagen 3 (col 3) (sense 5′-TTA TAA ACC AAC CTC TTC CT-3′ and antisense 5′-TAT TAT AGC ACC ATT GAG AC-3′) and GAPDH (sense 5′-TCA CCA TCT TCC AGG AGC GA-3′ and antisense 5′-CAC AAT GCC GAA GTG GTC GT-3′).

The RT-PCR mixture contained 5 µl of the inverse transcription product (cDNA) diluted 1∶20, 10 µl of iQ SYBR Green Supermix (Bio-Rad Laboratories, Hercules, CA, USA) and 1 µl (6 µM) of each primer in a final reaction volume of 20 µl. The RT-PCR was performed in a StepOnePlus Real-Time PCR System (Applied Biosystems, Foster City, California, USA). The samples were subjected to an initial stage of 10 minutes at 95°C. The conditions for cDNA amplification were as follows: 40 cycles of 95°C for 15 s, 60°C (col 1 and 3) or 55°C (GAPDH) for 30 s and 72°C for 1 minute. The products were subjected to 2% agarose gel electrophoresis, stained with a SYBR Green II RNA gel stain (Invitrogen) and visualized by UV light. The gene expression was normalized against the expression recorded for the constitutive gene glyceraldehyde 3-phosphate-dehydrogenase (GAPDH).

### Immunofluorescence Microscopy/collagen Expression

The collagen content was detected by immunofluorescence. The monoclonal antibodies anti-collagen I (Sigma, St. Louis, MO, USA) and anti-collagen III (Medicorp, Montreal, Canada) were used as primary antibodies. A secondary antibody conjugated with rhodamine was used in the study. The negative controls were subjected to 3% BSA instead of the primary antibody. The cell nuclei were counterstained with DAPI. The samples were examined under a Leica SP5 confocal microscope (Leica Microsystems, Wetzlar, ***Germany***) to detect fluorescence.

### Immunohistochemistry/macrophage Response

For immunohistochemistry, a specific monoclonal antibody to rabbit macrophages, RAM-11 (DAKO M-633, USA), was used as primary antibody. The antigen-antibody reaction was detected by alkaline phosphatase avidin-biotin procedures. The chromogenic substrate contained alpha-napthol and fast-red. The nuclei were counterstained with acid hematoxylin. RAM-11-labeled macrophages were quantified by performing counts in 20 microscopic fields (×20) for each biomaterial.

### Biomechanical Strength

To determine the biomechanical strength and modulus of elasticity of the meshes after implant, strips of the different biomaterials (1.5 cm wide and 5 cm long) including the mesh and the infiltrated host tissue, were analyzed using an INSTRON 3340 testing system (static load 500N) (Instron Corp., UK). The crosshead speed was 5 cm/minute, and the recording speed was 2 cm/minute.

All of the tests were conducted immediately after the animals were sacrificed at the different study times.

### Statistical Analysis

The statistical analysis was performed using the Graph Pad Prism 5 package (GraphPad Soft-ware, Inc., La Jolla, CA, USA). The percentages of mRNA expression and RAM-11 positive cells, as well as the measurements of biomechanical strength, were compared among the three study groups using the Mann-Whitney U test. The results were expressed as the mean ± SEM. The level of statistical significance was set at *P*<0.05.

## Results

### Macroscopic Analysis

At 14 days, seroma formation was observed in three of the six *Bio-A* implants. At 30 days, the biomaterial presented areas of degradation. By 90 days, the prosthesis had almost completely disappeared. After 6 months, the area of the implant was covered by newly formed tissue and appeared to be partially distended.

No seromas were observed in *Tuto*, but signs of degradation of this biomaterial were observed in 2 of the samples at 14 days. 30 days after implant at least half of the samples showed signs of degradation and/or disinsertion of the biomaterial. By 180 days, the prosthesis had completely disappeared, and the implant area appeared to be distended.

**Figure 2:**
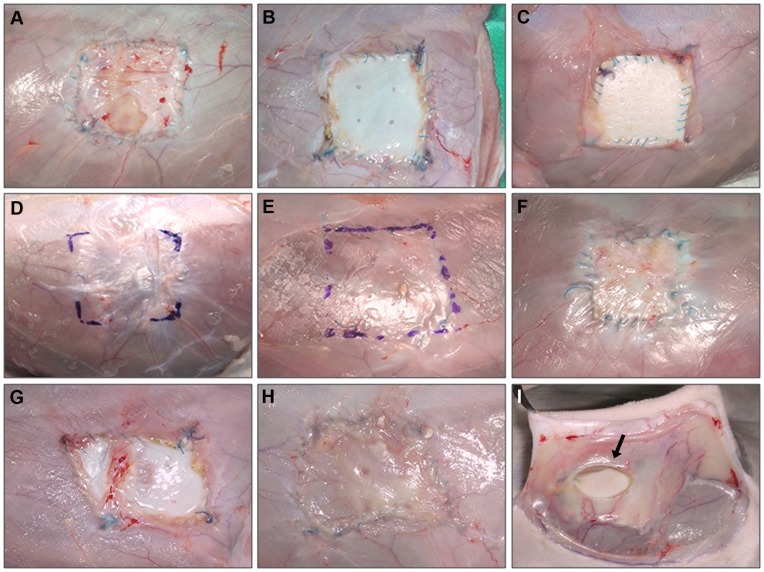
Macroscopic biomaterial images. *Bio-A* implants after 14 days (A) and 30 days postimplantion (D). *Tuto,* 14 days (B) and 30 days (E). *St*, 14 days (C) and 30 days (F). Partial degradation of *Tuto* at 14 (G) and 30 days (H). *St* encapsulation (arrow) at 30 days post-implantation (I).

In the implants using *St*, seromas were observed in two of the six implants at 2 weeks and in one at 30 days. No signs of prosthetic degradation were observed at any of the timepoints (Fig. 2).

### Morphology

**Figure 3:**
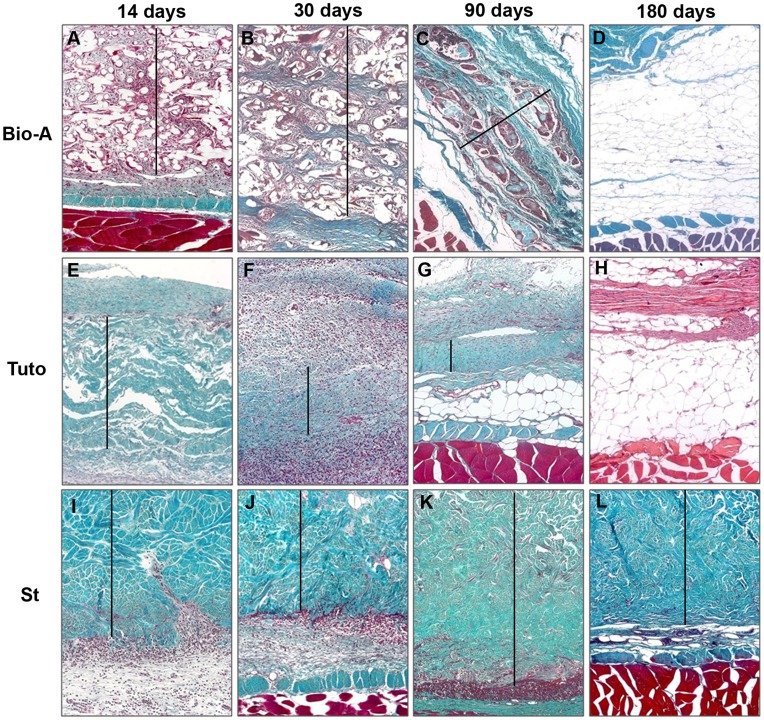
Light microscopy images. Tissue integration and prostheses degradation in the different timepoints (100×). *Bio-A* (A–D) and *Tuto* (E–H) showed a gradual infiltration of host tissue and was completely degraded by 180 days post-implantation. *St* exhibited material encapsulation, without signs of degradation and low cell infiltration at 180 days (I–L). (– Prosthesis).

At 14 days and 30 days (Fig. 3A and B), the filaments of the *Bio-A* prostheses were surrounded by an intense inflammatory reaction and loose connective tissue. At 30 days, the polymer showed initial signs of degradation, and the neoformed tissue was growing denser. At 3 months (Fig. 3C), an evident degradation of the filaments was observed; these areas were occupied by connective tissue composed of large bundles of collagen fibers. The filaments appeared to be surrounded by inflammatory cells. By 6 months post-implantation (Fig. 3D), the prosthetic components–as well as the inflammatory cells–had disappeared, and the connective tissue had been almost completely substituted by adipose tissue.

At 2 weeks (Fig. 3E), the *Tuto* prosthesis appeared to be infiltrated by a small population of receptor-tissue cells throughout its thickness. A moderate inflammatory response occurred, which significantly increased at 30 days (Fig. 3F). This response decreased significantly at 90 days post-implantation (Fig. 3G), along with a significant degradation of the prosthesis and a decrease in its thickness. By 180 days (Fig. 3H), as in the case of *Bio-A*, the biomaterial was completely reabsorbed and replaced by adipose tissue and areas of dense connective tissue.

The *St* implant was initially observed to be surrounded by a capsule of highly vascularized connective tissue and to present an intense inflammatory reaction (Fig. 3I,J). 90 days after implant, the neoformed connective tissue and the inflammatory cells colonized the inferior and superior thirds of the bioprosthesis (Fig. 3K). No evident signs of reabsorption of the collagen lamina were observed at the different timepoints. Subsequently, 180 days after implant, a significant decrease in the inflammatory reaction was observed (Fig. 3L), compared with the previous timepoints (14, 30 and 90 days) where the inflammatory reaction was more intense.

### Real-time RT-PCR

**Figure 4:**
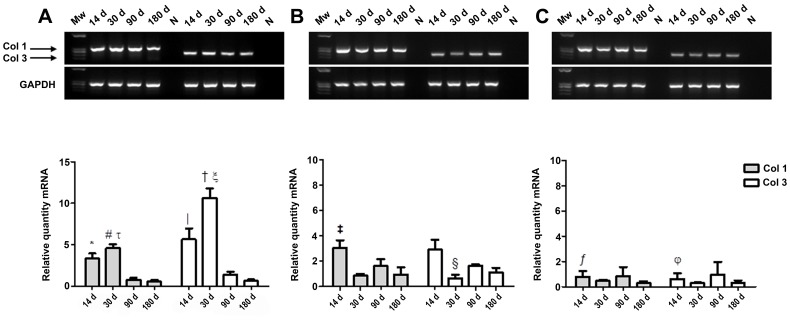
Collagen 1 and 3 mRNA expression determined by RT-PCR. Agarose gel product and relative mRNA quantity of *Bio-A* (A), *Tuto* (B) and *St* (C) after 14, 30, 90 and 180 days post-implantation. The results are expressed as the mean ± SEM of three experiments. Gene expression was normalized with the GAPDH gene. A) *Bio-A*: Collagen (Col) 1: *, vs. 90 days (*P*<0.05) and 180 days (*P*<0.01); #, vs. 90 days (*P*<0.05) and 180 days (*P*<0.01); τ, vs. *Tuto* (*P*<0.05) and *St* (*P*<0.01) at 30 days. Col 3: |, vs. 30 days and 90 days (*P*<0.05) and 180 days (*P*<0.001); †, vs. 90 days (*P*<0.05) and 180 days (*P*<0.001); ξ, vs. *Tuto* (*P*<0.05) and *St* (*P*<0.01) at 30 days. B) *Tuto*: Col 1: ‡, vs. 30 days (*P*<0.05). Col 3: §, vs. 14 and 90 days (*P*<0.05). C) *St*: Col 1: ƒ, vs. *Bio-A* and *Tuto* at 14 days (*P*<0.01). (N = negative control; Mw = molecular weight markers).

We studied the mRNA expression of collagens 1 and 3 in the neoformed tissue (Fig. 4). The synthetic mesh exhibited a higher expression of both collagens versus the biological prostheses. The behavior of the immature (collagen 3) and mature (collagen 1) was similar and independent of the type of prosthesis used.

The pattern of mRNA expression for both types of collagen was similar in the two bioprostheses but completely different in the *Bio-A* implant. *Bio-A* exhibited a significant increase in collagen 1 and 3 mRNA expression at 14 and 30 days, that significantly decreased at later timepoints. However, the biological meshes yielded two peaks demonstrating the higher expression of both types of collagen at 14 and 90 days.

For *Tuto*, the expression of collagen 1 and 3 at 14 days was significantly higher than at 30 days, and the collagen 3 mRNA demonstrated differences between 30 and 90 days. For *St*, we did not observe any significant difference over time.

When the different types of meshes were compared at 14 and 30 days, *Bio-A* exhibited a greater collagen 1 and 3 mRNA expression compared with the two other biological prostheses. Significant differences were observed at 14 days in both types of collagens when *Bio-A* and *St* were compared and when *St* was compared with *Tuto*. At 30 days, significant differences were observed in both types of collagens when the synthetic mesh was compared with the two bioprostheses. Any significant differences were observed at 90/180 days.

### Immunofluorescence Microscopy/collagen Expression

To identify the neoformation of native collagen in the bioprostheses, we generated differential interference contrast (DIC) images, superimposing the immunofluorescence confocal images. Consequently, the new collagen was labeled by red fluorescence, and the collagen or synthetic prostheses appeared translucent.

**Figure 5:**
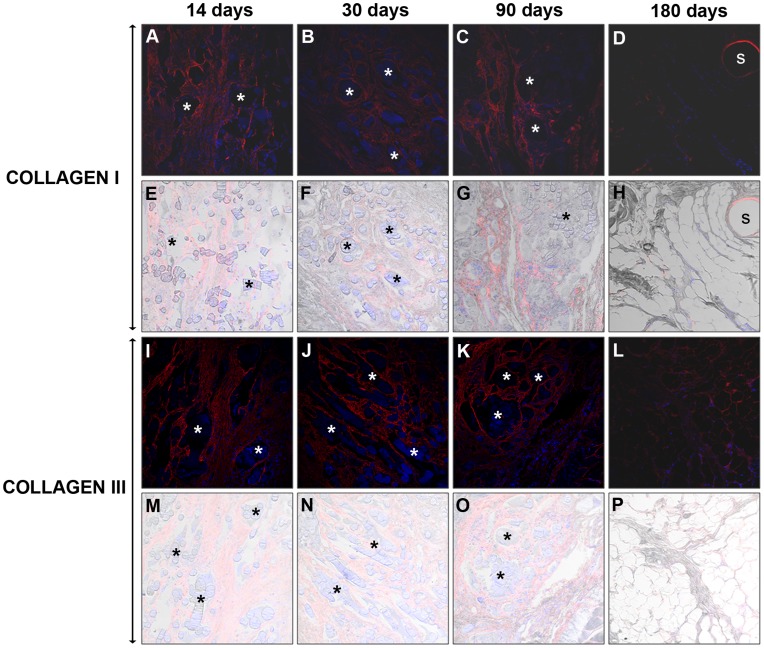
Immunodetection of neoformed collagen I and III in *Bio-A*®. Mature Collagen I (A–H) and immature Collagen III (I–P) immunofluorescence at 14, 30, 90 and 180 days post-implantation. The neoformed collagen appears in red and the cell nuclei (stained with DAPI) appear in blue. The DIC images that identify the biomaterial appear translucent (E–H and M–P). Confocal light microscopy (200×). (* Synthetic mesh).

In the *Bio-A*, the expression of mature collagen was first observed at 14 days post-implantation, around the filaments of the prosthesis. The labeling was maintained between 30 and 90 days, but a decrease occurred at 180 days, when the adipose tissue occupied the greatest area of the implant. The intensity of the collagen III labeling was similar at all of the timepoints and was localized to the tissue invading the prosthesis. At 180 days, the collagen III was mainly found around the adipose tissue (Fig. 5).

In *Tuto*, the intensity of the collagen I labeling was greater at the earlier timepoints and subsequently decreased. It was confirmed that the unimplanted prosthesis exhibited an immunoreactivity for collagen I similar to that observed 14 days post-implantation, which prevented the differentiation of the prosthetic collagen I from the newly formed native collagen.

**Figure 6:**
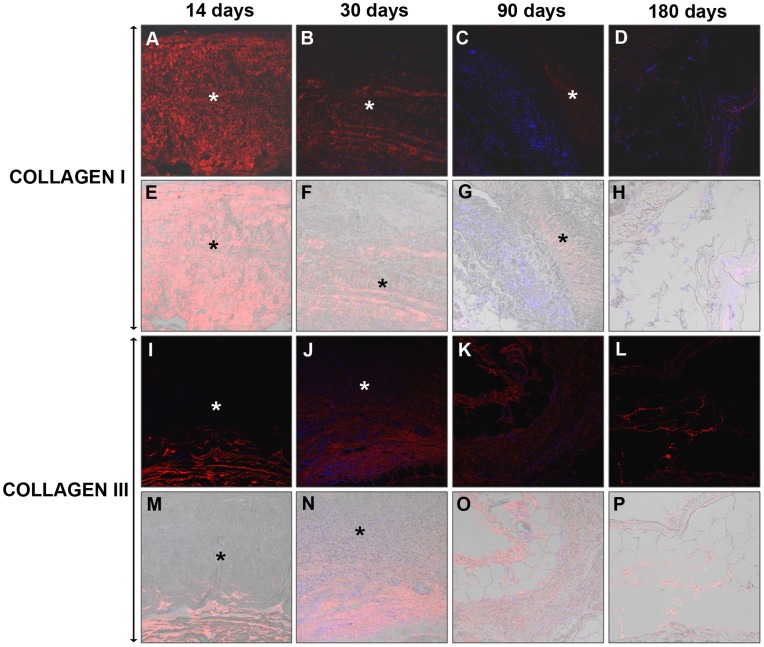
Immunodetection of neoformed collagen I and III in *Tutomesh*®. Mature Collagen I (A–H) and immature Collagen III (I–P) immunofluorescence at 14, 30, 90 and 180 days post-implantation. The neoformed collagen appears in red and the cell nuclei (stained with DAPI) appear in blue. The DIC images that identify the biomaterial appear translucent (E–H and M–P). Confocal light microscopy (200×). (* Biomesh).

Compared with collagen I, the expression of collagen III was predominant, as determined by the more intense labeling of the latter at all of the timepoints (Fig. 6).

**Figure 7:**
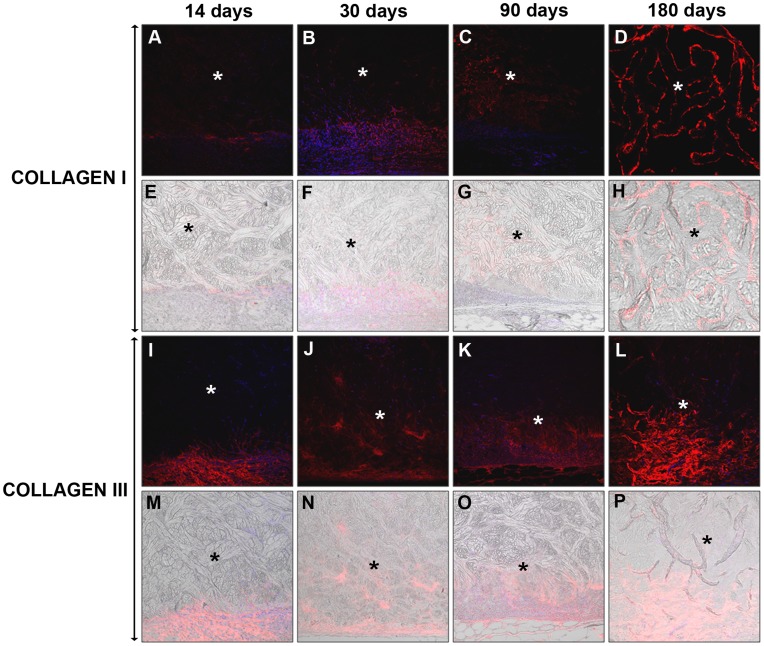
Immunodetection of the neoformed collagen I and III in *Strattice*®. Mature Collagen I (A–H) and immature Collagen III (I–P) immunofluorescence at 14, 30, 90 and 180 days post-implantation. The neoformed collagen appears in red and the cell nuclei (stained with DAPI) appear in blue. The DIC images that identify the biomaterial appear translucent (E–H and M–P). Confocal light microscopy (200×). (* Biomesh).

In *St*, collagen I expression increased in the long term. At 14 days, the fluorescence was faint and limited to the capsule of tissue that formed between the prosthesis and the muscle. No labeling was observed in the interior of the prosthesis. At day 30, the labeling was observed in certain areas of the interior of the prosthesis that had been infiltrated by the neoformed tissue; moreover, there was increased labeling of the capsular tissue. By 180 days, the fluorescence intensity had increased inside the prosthesis. The expression of collagen III was evident at all of the timepoints and followed a pattern of expression that was similar to that of collagen type I (Fig. 7).

### Immunohistochemistry/macrophage Response

Both collagen meshes elicited a similar expression of macrophages, which decreased over time. However, the synthetic mesh exhibited a different pattern of expression, which remained at high and homogeneous levels until 90 days post-implantation, then decreased significantly thereafter. The *Bio-A* synthetic prosthesis presented a significantly greater percentage of positive cells compared with the biological prostheses (except for the *Tuto* at 14 days). The active macrophages primarily formed foreign-body giant cells around the absorbable filaments.

**Figure 8:**
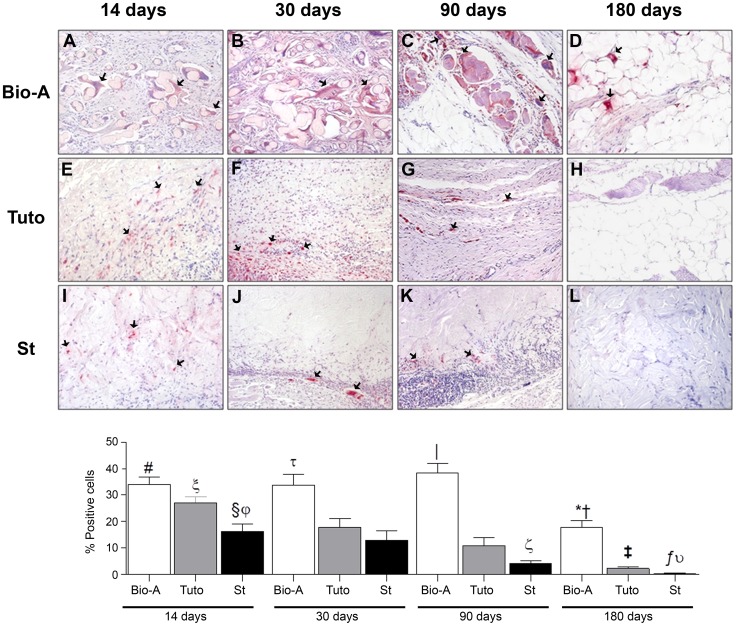
Foreign-body reaction of the different meshes. Immunohistochemical labeling of rabbit macrophages (red color, arrows) using the RAM-11 monoclonal antibody (200×) (top panel): *Bio-A* (A–D), *Tuto* (E-H) and *St* (I–L). Percentage of positive cells for the RAM-11 antibody in the different prostheses after 14, 30, 90 and 180 days post-implantation (bottom panel). The results were expressed as the mean ± SEM. A) *Bio-A*: *, vs. 14 days and 90 days (*P*<0.001) and 30 days (*P*<0.01); #, vs. *St* (*P*<0.001) at 14 days; τ, vs. *Tuto* and *St* (*P*<0.01) at 30 days; |, vs. *Tuto* and *St* (*P*<0.001) at 90 days; †, *Tuto* and *St* (*P*<0.001) at 180 days post-implantion. B) *Tuto*: ξ, vs. 30 days (*P*<0.05) and 90 days (*P*<0.001); ‡, vs. 14 days and 30 days (*P*<0.001) and 90 days (*P*<0.01). C) *St*: §, vs. 90 days (*P*<0.001); ƒ, vs. 14, 30 and 90 days (*P*<0.001); φ, vs. *Tuto* (*P*<0.01) at 14 days; ξ, vs. *Tuto* (*P*<0.05) at 90 days; υ, vs. *Tuto* (*P*<0.001) at 180 days post-implantation.

Both biological meshes exhibited a short-term localization of positive cells to the inflammatory tissue that formed between the prosthesis and the recipient muscle; at later timepoints, these cells formed visible colonies within the prosthesis. Subsequently, this response significantly decreased, almost disappearing in the case of *St* at 180 days (Fig. 8).

### Biomechanics

**Figure 9:**
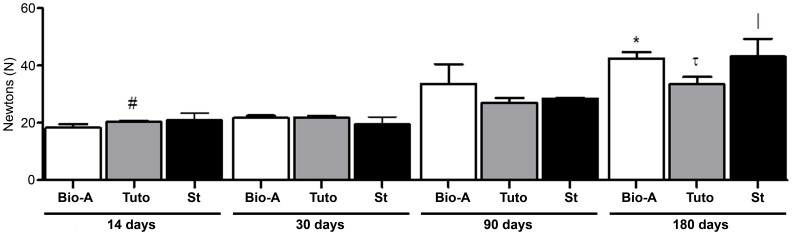
Biomechanical strength of the different meshes. The results (Newtons) were expressed as the mean ± SEM at 14, 30, 90 and 180 days post-implantation. *Bio-A*: *, vs. 14 days and 30 days (*P*<0.01). *Tuto*: #, vs. 90 days (*P*<0.01); τ, vs. 14 days and 90 days (*P*<0.05) and 30 days (*P*<0.01). *St*: |, vs. 14 days and 30 days (*P*<0.05).

At the earliest timepoints (14 and 30 days), the resistance to breakage of the different prostheses was similar. A slight increase was observed at 90 days, with no significant differences prior to this timepoint. The greatest gain in biomechanical resistance was reached at 180 days, when resistance to breakage was significantly increased in *Bio-A* (p<0.01) and *St* (p<0.05) implants at 14 and 30 days (Fig. 9).

## Discussion

The use of collagen-derived prostheses for elective hernia repair are feasible alternatives in contaminated abdominal defects for which synthetic prostheses are contraindicated [8]. However, the most important limitations to the clinical use of collagen-derived prostheses are their costs and the cultural groups of patients in whom they may be implanted [9].

The goal of using these biomaterials in tissue-engineering is to achieve not only repair but also tissue regeneration [1]. To this end, once implanted, the biomaterials promote angiogenesis and the formation of new tissue, which are processes involving the stimulation of growth factors and the synthesis of extracellular matrix elements.

The ideal characteristic of biomeshes is that they do not rapidly degrade, remaining stable until they are gradually and fully incorporated into the recipient tissue. For this function, it is necessary for the triple-helix links that constitute the collagen molecule to be efficient; otherwise, the mechanical firmness will be compromised. The success of the repair would thus depend on the balance between the tissue-regeneration processes and the degradation of the prosthesis.

Resembling these collagen-derived biological prostheses, the synthetic prosthesis (*Bio-A*) used in this study is formed by a PGA and TMC copolymer. This material can undergo a progressive biodegradation through hydrolytic and enzymatic processes that do not leave any permanent residues in the body.

Various investigators have attempted to demonstrate the possible advantages of biomeshes over synthetic prostheses. A recently published *in vitro* study [10] reveals that human mononuclear cells are activated by porcine crosslinked bioprostheses, which induce a greater expression of cytokines compared with those noncross-linked. This finding has been attributed to the processing methods and/or the degree of collagen cross-linking.

*St* belongs to a new generation of porcine bioprostheses that are derived from the dermis and are processed without chemical cross-links; in it the galactose-alpha 1,3 antigen–which is the greatest elicitor of the immune responses associated with acellular xenografts–has been enzymatically eliminated [11], [12]. When implanted into the ventral hernial defects of non-human primates, *St* does not provoke a xenogeneic immune response [12]. This fact was confirmed in the present study, in which *St* elicited the lowest macrophage response, despite being derived from a different animal species.

A recent study [13] has demonstrated that cross-links materials may be more durable during the remodeling process, as suggested by the progressive and significant thinning and weakening of *St*. By contrast, we found [14] that the behavior of *St* was similar to that of the other prostheses with similar thickness and cross-links (*Collamend®*/*Permacol®*) however, over time, we observed a greater cellular infiltration and deposits of neoformed collagen for St. Consistent with our results, the Butler group [15] has tested *Collamend®* and *St* and affirms that these prostheses exhibit rapid tissue and vascular infiltration, indicating the clinical advantages of an abdominal reconstruction that does not compromise the resistance to the implant’s traction area. However, compared with bioprostheses of human origin, *Collamend®* and *St* exhibit greater cellular and vascular infiltration [16] and provide stronger support [17].

*Tuto*, is a non-crosslinked prosthesis created from bovine pericardium that has been used in the repair of multiple structures, such as the pericardium, various types of hernias and the pelvic floor.

An investigation using bovine pericardium [2] in a rat subcutaneous implant has revealed how the degree of crosslinkage determines the index of prosthetic degradation and thus significantly affects the pattern of tissue regeneration. Another experimental study [18] of total abdominal defects (also in rats) has revealed that the bovine pericardium and ePTFE elicits a minimal foreign-body reaction and provides adequate mechanical resistance at 2 weeks post-implantation. These results are consistent with our study, in which both collagen meshes elicited a similar macrophage response that decreased over time. Clinical studies of contaminated tissues [19], [20] have demonstrated that the acellular bovine pericardium may be a useful tool for these situations.

Despite the good behavior showed by biological prostheses, several clinical complications like evisceration, disintegration, poor mesh integration, infection or seroma, associated with xenograft biologic mesh implantation in abdominal wall reconstruction, have been described [5]. A systematic review recently published, has stated that wound infection and seroma formation are the most common postoperative complications related to biological prostheses implantation [21].

*Bio-A*, is a biodegradable material that has been used for applications other than the abdominal wall, such as anal fistulas [22], [23]. Regarding hernia repair, a clinical pilot study of 10 patients [7] demonstrated the efficiency of *Bio-A* in this type of surgery. Other authors [24] have reported the suitability of this material in a single clinical case that involved the repair of a large inguinal hernia.

In our experimental study, *Tuto* and *Bio-A* macroscopically exhibited a rapid degradation, being complete between 90 and 180 days post-implantation. This did not occur with the *St* implants, which were significantly more stable over time, remaining intact at 6 months post-implantation. Areas of tissue “relaxation” at the level of the defects could be observed in *Tuto* and *Bio-A*, without herniation of the intra-abdominal contents, that have been reported in clinical practice [25].

In our experimental study, the noncross-linked collagen implants exhibited the early overexpression of the collagen 1 and 3 genes, consistent with a previous study [14]. Two expression peaks occurred at 14 and 90 days. However, *Bio-A* demonstrated a completely different pattern of expression, with a significant increase in collagen 1 and 3 mRNA at 30 days, whereas this expression significantly decreased at later timepoints.

Regarding the inflammatory reaction, *Bio-A* elicited a significantly greater macrophage response until 90 days post-implantation, compared with the collagen implants. This finding was inconsistent with another study [6] that has demonstrated an attenuated inflammatory response in *Bio-A* compared with a biomesh of human origin.

From the biomechanical point of view, the existing areas of “relaxation” at the implant sites did not correlate with the final mechanical resistance that was obtained. This result indicated a progressive increase in resistance to breakage in each of the prosthetic materials throughout the timepoints in the study. Most likely, in the materials with the greatest biodegradation, the tissue support of the implant region (partial defect) restricts the exact analysis of the resistance of the various materials to breakage, which should obviously be considered when evaluating these parameters.

In summary, we can affirm that prostheses of the absorbable laminar type are among the newly available tools within a surgeon’s therapeutic arsenal. They provide a scaffold upon which the host’s fibroblasts can form well organized and adequately vascularized new connective tissue. However, these prostheses are still inadequate with respect to their precise timepoints of biodegradation, which fundamentally affects the repair process.

In the present study, *St* yielded optimal results during the repair process, with a progressive collagenization and minimal foreign-body reactions. We considered that the slower degradation of *Bio-A*, which is a completely polymeric prosthesis, could offer excellent reparative results compared with the prostheses of biological origin, particularly regarding the cost-benefit of the biomaterial.

Ultimately, we conclude that in the present study, the *St* prostheses exhibited the optimal tissue behavior. However, additional and long-term studies of this biomaterial are necessary to corroborate its total degradation after generating new tissue within the implant area.
